# Characterization of denitrifying activity by the alphaproteobacterium, *Sphingomonas wittichii* RW1

**DOI:** 10.3389/fmicb.2014.00404

**Published:** 2014-08-06

**Authors:** Lynnie S. Cua, Lisa Y. Stein

**Affiliations:** ^1^Department of Environmental Sciences, University of CaliforniaRiverside, CA, USA; ^2^Department of Biological Sciences, Faculty of Science, University of AlbertaEdmonton, AB, Canada

**Keywords:** *Sphingomonas wittichii* RW1, nitrous oxide, nitrite reductase, nitric oxide reductase, nitric oxide dioxygenase, denitrification

## Abstract

*Sphingomonas wittichii* RW1 has no reported denitrifying activity yet encodes nitrite and nitric oxide reductases. The aims of this study were to determine conditions under which *S. wittichii* RW1 consumes nitrite (NO^−^_2_) and produces nitrous oxide (N_2_O), examine expression of putative genes for N-oxide metabolism, and determine the functionality of chromosomal (ch) and plasmid (p) encoded quinol-dependent nitric oxide reductases (NorZ). Batch cultures of wildtype (WT) and a *norZ*_ch_ mutant of *S. wittichii* RW1 consumed NO^−^_2_ and produced N_2_O during stationary phase. The *norZ*_ch_ mutant produced N_2_O, although at significantly lower levels (c.a. 66–87%) relative to the WT. Rates of N_2_O production were 2–3 times higher in cultures initiated at low relative to atmospheric O_2_ per unit biomass, although rates of NO^−^_2_ consumption were elevated in cultures initiated with atmospheric O_2_ and 1 mM NaNO_2_. Levels of mRNA encoding nitrite reductase (*nirK*), plasmid-encoded nitric oxide dioxygenase (*hmp*_p_) and plasmid-encoded nitric oxide reductase (*norZ*_p_) were significantly higher in the *norZ*_ch_ mutant over a growth curve relative to WT. The presence of NO^−^_2_ further increased levels of *nirK* and *hmp*_p_ mRNA in both the WT and *norZ*_ch_ mutant; levels of *norZ*_p_ mRNA compensated for the loss of *norZ*_ch_ expression in the *norZ*_ch_ mutant. Together, the results suggest that *S. wittichii* RW1 denitrifies NO^−^_2_ to N_2_O and expresses gene products predicted to detoxify N-oxides. So far, only *S. wittichii* strains within four closely related taxa have been observed to encode both *nirK* and *norZ* genes, indicating a species-specific lateral gene transfer that may be relevant to the niche preference of *S. wittichii*.

## Introduction

Denitrification is the sequential reduction of nitrate (NO^−^_3_) and nitrite (NO^−^_2_) to dinitrogen (N_2_) via the gaseous intermediates, nitric oxide (NO) and nitrous oxide (N_2_O) (Zumft, 1997). Respiratory denitrification is considered an anaerobic energy-generating metabolism; however, many bacteria can denitrify in the presence of O_2_ starting with NO^−^_3_ or NO^−^_2_ and terminating with N_2_O due to inhibition or absence of nitrous oxide reductase (Philippot et al., 2011; Stein, 2011; Chen and Strous, 2013). While some denitrifiers grow via hybrid respiration with O_2_ and NO^−^_3_ and/or NO^−^_2_, others respire N-oxides during late-log to stationary phase for detoxification and/or energy conservation, particularly under reduced O_2_ (Takaya et al., 2003; Stein, 2011; Chen and Strous, 2013). The collective role of denitrifying bacteria in transformation and release of highly reactive N-oxides is of critical importance because of the effects these molecules have on environmental and human health, atmospheric chemistry, and global warming (Fields, 2004; Galloway et al., 2008).

*Sphingomonas wittichii* RW1 was isolated from the River Elbe (Germany) as a model organism for studying the bioremediation of dioxin-containing compounds (Wittich et al., 1992; Wilkes et al., 1996; Yabuuchi et al., 2001; Keum et al., 2008). The complete genome sequence of *S. wittichii* RW1 revealed the presence of a single circular chromosome and two megaplasmids (Miller et al., 2010). Although not known to denitrify, *S. wittichii* RW1 encodes in its genome a copper-containing nitrite reductase (*nirK*) as the terminal member of a four-gene cluster with a NO-responsive NsrR regulator encoded upstream (Swit_1789-93). This gene cluster shares structural and sequence homology to ammonia- and nitrite-oxidizing bacteria in the *Nitrosomonas* and *Nitrobacter* genera, respectively (Cantera and Stein, 2007). *S. wittichii* RW1 also encodes a chromosomal (Swit_4614) and plasmid copy (Swit_5200) of quinol-linked nitric oxide reductase (*norZ*). NorZ is often expressed in non-denitrifying pathogenic bacteria for NO detoxification (Hendriks et al., 2000), but can also act alongside the terminal oxidase in the aerobic respiratory chain for energy conservation (Chen and Strous, 2013). The plasmid-encoded *norZ*_p_ (Swit_5200) is the terminal member of a four-gene cluster; the first member of which encodes a nitric oxide dioxygenase (*hmp*_p_; Swit_5203) with predicted function in NO oxidation to NO^−^_3_ or NO reduction to N_2_O depending on O_2_ concentration (Bonamore and Boffi, 2008). Nitric oxide dioxygenases are present in both denitrifying and non-denitrifying microorganisms to combat nitrosative and oxidative stresses (Bonamore and Boffi, 2008; Forrester and Foster, 2012). Although nitric oxide dioxygenases are usually conserved members of the NO-controlled NsrR transcriptional regulon in bacteria (Rodionov et al., 2005), the plasmid-encoded gene cluster in *S. wittichii* RW1 that includes both NorZ and nitric oxide dioxygenase is preceded by a CDS for the NO-responsive NnrR transcriptional regulator (Swit_5204). Aside from Swit_5203, *S. wittichii* RW1 encodes three other putative *hmp* genes, the plasmid-encoded Swit_5299 and the chromosomal Swit_1434 and 3173. A comparison of 51 genome-sequenced sphingomonad strains (encompassing the *Sphingomonas, Sphingobium, Novosphingobium*, and *Sphingopyxis* genera) by BLAST searches through the Integrated Microbial Genomes database (http://img.jgi.doe.gov) revealed that only the two strains of *S. wittichii* (RW1 and DP58) encode the complete *nirK* gene cluster, whereas eight sphingomonad genomes encode either one or two copies of *norZ* and 17 genomes encode one or more *hmp* genes whose translated sequences share >60% protein identity to Swit_5203. Hence, the potential for spingomonad bacteria to transform nitrogen oxides appears to be fairly restricted.

Previous studies in *Neisseria* and *Synechococcus* demonstrated that disruption of *norZ* expression resulted in increased NO sensitivity, diminished NO consumption and N_2_O production, and decreased growth under anoxia (Householder et al., 2000; Busch et al., 2002). Interestingly, *Ralstonia eutropha* H16 also possesses two independent quinol-linked nitric oxide reductases. Deletion of either gene in *R. eutropha* H16 resulted in no phenotypic change under aerobic or anaerobic growth at the expense of NO^−^_3_ or NO^−^_2_ (Cramm et al., 1997). Therefore, in the present study we tested the hypothesis that the *norZ* genes in *S. wittichii* RW1 are similarly isofunctional.

The overarching hypothesis of the present study is that *S. wittichii* RW1 reduces NO^−^_2_ to N_2_O and thus can be classified as a denitrifying strain. The ability of *S. wittichii* RW1 to denitrify from NO^−^_3_ was not investigated as the genome of *S. wittichii* RW1 encodes only the alpha subunit of assimilatory nitrate reductase (Swit_1709) and no features of dissimilatory nitrate reductases. Furthermore, this strain tested negative for reduction of NO^−^_3_ to NO^−^_2_ (Yabuuchi et al., 2001). There is no identifiable sequence in the genome with similarity to nitrous oxide reductase; hence, this strain is predicted to denitrify only NO^−^_2_ to N_2_O. To provide support for *S. wittichii* RW1 as a denitrifier, objectives were to: (a) determine whether and when *S. wittichii* RW1 produces N_2_O at the expense of NO^−^_2_ (b) investigate the regulation of putative N-oxide metabolism genes in response to varying NO^−^_2_, and (c) determine whether the chromosomal- and plasmid-encoded *norZ* genes in *S. wittichii* RW1 are isofunctional.

## Materials and methods

### Culture maintenance

*Sphingomonas wittichii* RW1 was provided as a gift from Dr. Rolf Halden. Cultures were grown in 5 mL Luria-Bertani Broth (LB) in sterilized 15 mL capped-polystyrene tubes in a rotary shaker (180 r.p.m.) at 28°C. Cultures were periodically streaked and grown on LB agar plates for single colony isolation to maintain culture purity.

### Construction of *norZ*_ch_ mutant of *S. wittichii* RW1

The region from bp 203 to 776 of the *norZ*_ch_ gene was PCR-amplified from *S. wittichii* RW1 genomic DNA with primers 203F 5′ aactggaacaggccgatg 3′ and 776R 5′ cgatcgccttcatcttcg 3′ to make use of an internal BclI restriction site [Primer3 Input 0.4.0 software (Rozen and Skaletsky, 2000)]. The amplification product was purified and ligated to the pGEM®-T Vector according to manufacturers' instructions (Promega Corp., Madison, WI). The ligation mixture was transformed into *dam*^−^/*dcm*^−^ competent *E. coli* cells (New England BioLabs Inc., Ipswich, MA) and transformants were selected via blue-white screening on LB agar plates containing 0.5 mM IPTG, 80 μg/mL X-Gal, and 100 μg/mL ampicillin. Plasmids from positive transformants were purified using Wizard® *Plus* SV Minipreps DNA Purification System kit (Promega Corp., Madison, WI) and digested with the BclI restriction enzyme (New England BioLabs Inc., Ipswich MA). The digest was run on a 0.8% agarose gel and linearized vector was gel-purified using the Wizard® SV Gel and PCR Clean-Up System kit (Promega Corp., Madison, WI).

A gentamycin-resistance cassette (871 bp) was digested from the pUCGM vector (gift from N. Hommes) using the BamHI restriction enzyme (New England BioLabs Inc., Ipswich MA). The digest was gel-purified from a 0.8% agarose gel and ligated to the previously BclI-digested pGEM-T-*norZ* vector. The ligation mixture was transformed into competent *E. coli* JM109 cells. Transformed cells were plated onto LB agar containing 100 μg/mL ampicillin and 10 μg/mL gentamycin. Positive transformants were verified by PCR and Sanger sequencing using the BigDye Terminator Cycle Sequencing Kit (Applied Biosystems, Foster City USA). Plasmids containing the correct inserts were purified as described above and electroporated into *S. wittichii* RW1 cells using an *E. coli* Pulser™ Transformation Apparatus (BioRad Laboratories, Hercules, CA). Competent *S. wittichii* RW1 cells were prepared by harvesting in exponential phase, washing three times with 20 mL ice-cold and nuclease-free water, washing twice with 2 mL ice-cold 10% glycerol, and resuspended in 10% glycerol to a final volume of 100 μL. Electroporated cells were plated onto LB agar containing 10 μg/mL gentamycin. The *norZ*_ch_ mutant strain was checked by PCR using additional primers: 45F 5′ agagacccaggaccacgac 3′, 854R 5′ tcaccgtcatggaatattgg 3′, pUCGM173F 5′ tgcctcgggcatccaagcagca 3′, pUCGM514R 5′ gagagcgccaacaaccgcttct 3′ and pUCGM519F 5′ cttacgttctgcccaggttt 3′. PCR products were purified and validated by Sanger sequencing. The *norZ*_ch_ mutant strain was maintained on LB media with 50 μg/mL gentamycin.

### Growth experiments

*S. wittichii* RW1 wildtype (WT) and *norZ*_ch_ mutant cells from exponentially growing cultures were inoculated into LB media (500 μL into 100 mL) containing 0, 0.3, or 1 mM NaNO_2_ into glass serum bottles (160 mL), which were then crimp-sealed with rubber septa and aluminum seals. Incubations of *norZ*_ch_ mutant cells contained 50 μg/mL gentamycin. Triplicate incubations of each control condition included the same concentrations of NaNO_2_ plus: (1) heat-inactivated cells, (2) no cells, or (3) live cells in bottles purged of O_2_ by sparging the medium with N_2_. All control incubations were treated identically to the experimental incubations to determine whether chemical decomposition of NO^−^_2_ contributed to NO^−^_2_ loss or N_2_O accumulation. Gas headspace (60 mL) was either left unchanged (atmospheric O_2_) or, for WT cells, sparged with N_2_ and injected with pure O_2_ prior to inoculation (ca. 3% O_2_ in gas headspace as validated by gas chromatography; GC-TCD, Shimadzu, Kyoto, Japan; Molecular Sieve 6A column, Alltech, Deerfield IL). Experimental and control bottles were incubated in a rotary shaker (180 r.p.m.) at 28°C. Starting at *t* = 0 h, 2 mL samples were extracted every 4 h using a sterile 1 mL needle and syringe. Growth was determined by measuring OD 600 nm using a Spectronic 20 Genesys spectrophotometer (Thermo Fisher Scientific, Inc., Waltham, MA). Cells were immediately treated with 500 μL RNAprotect™ Bacteria Reagent (Qiagen, Valencia, CA), and kept at −80°C. Experiments consisted of five independent trials performed on different days for both strains and under every condition.

### Nucleic acid extraction

Genomic DNA was isolated using the Wizard® SV Genomic DNA Purification System kit (Promega Corp., Madison, WI). Total RNA was extracted using the Aurum™ Total RNA Mini kit (Bio-Rad Laboratories, Hercules, CA). Nucleic acid concentration was determined using a NanoDrop® ND-1000 Spectrophotometer (Nanodrop Technologies, Inc., Wilmington, DE). DNA and RNA samples were kept at −20 and −80°C, respectively.

### Dot-blot hybridization

Gene-specific primers were designed from CDS's of selected genes from the *S. wittichii* RW1 genome sequence (Genbank accession: CP000699 to CP000701) using Primer3 Input 0.4.0 software (Rozen and Skaletsky, 2000) (Table 1). PCR reactions included standard reagents for Taq polymerase and genomic DNA as template in 25 μL reactions (Sambrook and Russell, 2001). Thermal cycler (iCycler, BioRad, Hercules, CA) amplification conditions were: 95°C for 5 min, 30 cycles of 95°C for 40 s, 55°C for 40 s and 72°C for 50 s, with an additional extension cycle of 72°C for 7 min. PCR products were checked by agarose gel (1%) to verify single products of appropriate size. Amplification products were purified using the Wizard® SV Gel and PCR Clean-Up System kit (Promega Corp., Madison, WI). Amplification products were labeled using the Prime-a-Gene labeling system (Promega Corp., Madison, WI) with [α-^32^P]-dCTP (3000 Ci mmol^−1^; Perkin-Elmer Inc., Waltham, MA) and random hexamers. The dynamic range of detection for each probe was tested using a concentration series of specific mRNA from 0.1 to 3 μg from control incubations (0 mM NaNO_2_). The r^2^ values for the slope of hybridization intensity/μg mRNA was from 0.94 to 1.0 for all probes.

**Table 1:** **Primers used to generate probes for RNA dot-blot hybridizations**.

**Locus Tag ^a^**	**Coding sequence ID**	**Enzyme commission number**	**F primer**	**R primer**	**Amplicon**
Swit_1793	NO-forming nitrite reductase *(nirK)*	EC:1.7.2.1	ctgaccgcgaaggaagtatc	catggtcgacgatcacattg	742 bp
Swit_5203 (p)	Nitric oxide dioxygenase *(hmp)*	EC:1.14.12.17	tcgagcttgtccacattctg	attgtctccccaaaccatga	210 bp
Swit_R0031	16S rRNA	untranslated	gtacaaggcctgggaacgta	tttatcgcctgaggatgagc	1159 bp
^*^Swit_5200 (p)	Nitric oxide reductase *(norZ)*	EC:1.7.5.2	ccaacgccaatactcaacct	cagcatttctacggcatcaa	513 bp
^*^Swit_4614 (ch)	Nitric oxide reductase *(norZ)*	EC:1.7.5.2	gtggtgcccgagaaatagag	gccagagcttctacggtgtc	703 bp

a *Significant difference between atmospheric and reduced O_2_ for wildtype (WT) cultures incubated with the same concentration of NaNO_2_*.

*(p), encoded on plasmid; (ch), encoded on chromosome*.

* *Swit_4614 and Swit_5200 share 54% amino acid sequence identity based on BLAST*.

*Primers were designed using Primer 3 Input 0.4.0 software (Rozen and Skaletsky, 2000) against the full CDS's from the complete genome sequence of Sphingomonas wittichii RW1, which includes a single circular chromosome and two megaplasmids (Genbank accession: CP000699–CP000701)*.

Two μg total RNA from each sample was blotted onto a Zeta-Probe® GT nylon membrane (Bio-Rad Laboratories, Hercules, CA) using a Minifold® microsample filtration manifold (Dot-Blot System, Schleicher & Schuell, Keene, NH) following the Zeta-Probe® protocol. Membranes were allowed to dry overnight and UV-crosslinked (FB-UVXL-1000, Fisher Scientific, Pittsburgh, PA). Prehybridization, hybridization, and washing of Zeta-Probe® nylon membranes were done according to manufacturer's instructions at 30°C. To allow re-probing, membranes were stripped of radioactivity by washing twice in a 0.1× SSC/0.5% SDS solution at 95–100°C for 20 min. All blots were hybridized to gene-specific probes to normalize hybridization signals to the 16S rRNA pool. Hybridization intensity was analyzed using a Typhoon Phosphorimager and Imagequant software (Amersham, Piscataway, New Jersey).

### Data analysis

Background and signal from non-specific binding was subtracted, after which the relative hybridization intensity of specific probes was normalized by dividing gene-specific signal by signal from 16S rRNA probe hybridizations. The fold difference in levels of mRNA for each gene and time point was determined by dividing hybridization intensities from dot blots of RNA extracted from NO^−^_2_ amended by those from unamended cultures. Student's *t*-test (*p* < 0.05) was performed to determine significant differences between treatments.

### Analytical measurements

Nitrite and ammonium were measured colorimetrically using standard methods (Clesceri et al., 1998). Nitrate was measured using a Standard Range Lab Nitrate Test kit (NECi, Lake Linden, Michigan). O_2_ and N_2_O were measured from the gas headspace of sample bottles by GC-TCD (Shimadzu, Kyoto Japan; Molesieve 5A and Hayesep Q columns, Alltech, Deerfield IL). Concentrations were determined by comparing to standard curves generated for each reagent and gas within the limits of detection.

## Results

### Effect of O_2_ and NO^−^_2_ on growth of WT and *NorZ*_ch_ mutant strains of *S. wittichii* RW1

**Figure 1:**
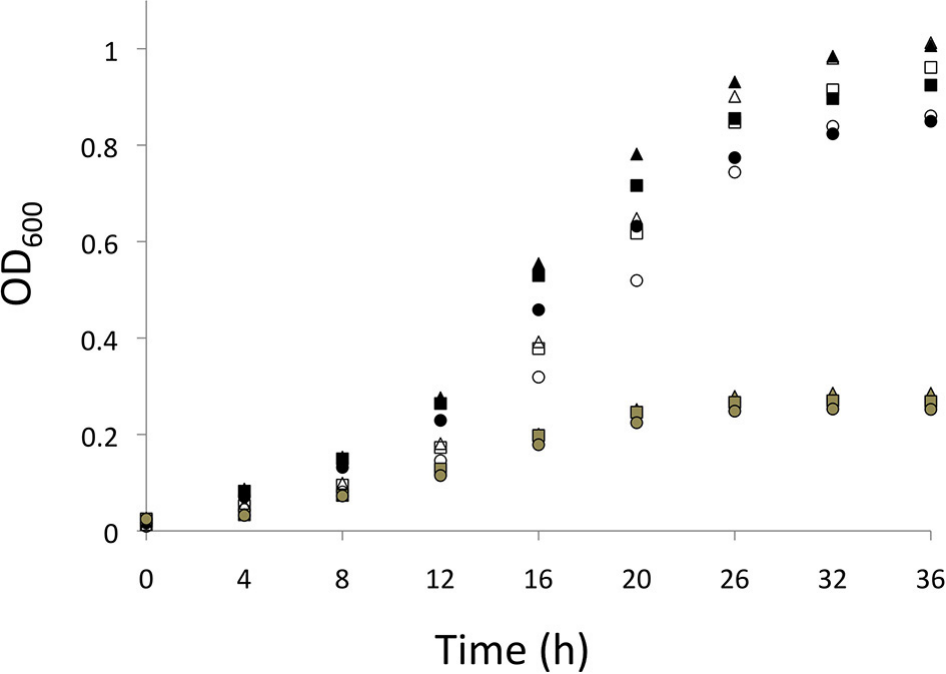
**Growth (OD_600_) measured over time (h) for *S. wittichii* RW1 WT cultures initiated at atmospheric O_2_ (black symbols) or reduced O_2_ (gray symbols), and for *norB*_ch_ mutant *S. wittichii* RW1 cultures initiated at atmospheric O_2_ (white symbols)**. Triangles = 0 mM added NaNO_2_, squares = 0.3 mM added NaNO_2_, and circles = 1 mM added NaNO_2_. Points on the growth curves represent averaged values from 5 independent experiments for each incubation condition.

*S. wittichii* RW1 is an aerobic heterotrophic bacterium; hence, the doubling time (calculated from 12 to 20 h growth) and final yields of non-mutagenized cells were significantly faster and higher, respectively, for cultures initiated under atmospheric compared to reduced (ca. 3%) O_2_ levels (Figure 1 and Table 2). Doubling times of the *norZ*_ch_ mutant were significantly shorter than those of the WT during exponential growth; thus, even though the *norZ*_ch_ mutant exhibited a longer lag phase, the cell density of the cultures were equivalent in stationary phase (Figure 1 and Table 2). The addition of NaNO_2_ to cultures initiated at atmospheric O_2_ only significantly increased the doubling time of WT cultures, but significantly reduced the final yields of both WT and *norZ*_ch_ mutant cultures (Table 2).

**Table 2:** **Growth of WT and *norZ_ch_* mutant strains of *S. wittichii* RW1 at variable NaNO_2_ and O_2_ concentrations**.

**Variable in growth condition**	**Doubling time (h)**		**Yield (OD600 nm)**	
	**WT**	***norZ_ch_***	**WT**	***norZ_ch_***
**Atmospheric O_2_, no NaNO_2_**	5.3^a^^b^^c^	4.4^c^	0.98^a^^b^	0.98^b^
**Reduced O_2_, no NaNO_2_**	8.2^a^	N.D.	0.25^a^	N.D.
**Atmospheric O_2_, 0.3 mM NaNO_2_**	5.6^a^^b^^c^	4.4^c^	0.90^a^^b^	0.91^b^
**Reduced O_2_, 0.3 mM NaNO_2_**	8.5^a^	N.D.	0.25^a^	N.D.
**Atmospheric O_2_, 1.0 mM NaNO_2_**	5.5^a^^b^^c^	4.4^c^	0.82^a^^b^	0.84^b^
**Reduced O_2_, 1.0 mM NaNO_2_**	8.4^a^	N.D.	0.22^a^^b^	N.D.

Doubling times were calculated during exponential growth from 12 to 20 h. Final cell yields were reported at 32 and 20 h growth when initiated at atmospheric (ca. 22%) and reduced (ca. 3%) O_2_ levels, respectively. Values represent averages from 5 separate experiments. Statistically significant differences between groups were determined by Student's t-test at p < 0.05 and are designated as follows:

a *Significant difference between atmospheric and reduced O_2_ for wildtype (WT) cultures only*.

b *Significant difference between WT or norZ_ch_ mutant cultures incubated with NaNO_2_ relative to unamended controls*.

c *Significant difference between WT and norZ_ch_ mutant cultures grown under identical conditions. N.D., not determined*.

### Consumption of NO^−^_2_ and production of N_2_O

Cultures of WT and *norZ*_ch_ mutant *S. wittichii* RW1 were incubated in the presence of NaNO_2_ to assess whether expression of *norZ*_ch_ was required for aerobic denitrifying activity. Amounts of remaining NO^−^_2_, remaining O_2_, and headspace N_2_O levels were compared over stationary phase (Table 3). NO^−^_2_ was consumed nearly to completion in both WT and *norZ*_ch_ mutant cultures by 96 h incubation. Neither WT nor *norZ_ch_* mutant cultures consumed O_2_ to complete anoxia and headspace O_2_ levels remained largely stable following 72 h incubation, even with continuous shaking at 180 rpm. N_2_O was measurable in the gas headspace starting after 48 h of incubation and continued to accumulate proportionally with the amount of added NaNO_2_ (Table 3). The *norZ*_ch_ mutant cultures produced significantly less N_2_O than the WT cultures (66–87% of WT levels) at both NO^−^_2_ concentrations. Nitrate production was not observed, which would be an expected aerobic activity of Hmp. NH^+^_4_ concentrations also did not vary between treatment groups, which would be expected if *S. wittichii* RW1 reduced NO^−^_2_ directly to NH^+^_4_ and allowed its accumulation prior to assimilation (data not shown). N_2_ was not measured. Control incubations containing heat-inactivated cells, no cells, or live cells inoculated into bottles sparged of O_2_ with N_2_ gas showed no consumption of NO^−^_2_ and no production of N_2_O.

**Table 3:** **Consumption of nitrite and oxygen and production of nitrous oxide by wild-type and *norZ_ch_* mutant cultures of *S. wittichii* RW1 initiated at atmospheric oxygen headspace and with 0.3 or 1 mM NaNO_2_**.

**Time (h)**	**NO^−^_2_ remaining (mM)**				**%O_2_ remaining in the headspace**				**N_2_O produced (nmolOD^−1^)**			
	**WT**		***norZ_ch_***		**WT**		***norZ_ch_***		**WT**		***norZ_ch_***	
	**0.3 mM**	**1.0 mM**	**0.3 mM**	**1.0 mM**	**0.3 mM**	**1.0 mM**	**0.3 mM**	**1.0 mM**	**0.3 mM**	**1.0 mM**	**0.3 mM**	**1.0 mM**
48	0.24^a^^b^	0.78^a^	0.27^a^^b^	0.79^a^	7.24^a^^b^	7.62^a^	7.82^b^	7.55	2.66^a^^b^	3.19^a^^b^	1.76^a^^b^	2.40^a^^b^
72	0.01^a^	0.24^a^^b^	0.04^a^	0.33^a^^b^	4.50^b^	4.65	4.71^b^	4.63	10.30^a^^b^	34.68^a^^b^	8.80^a^^b^	24.99^a^^b^
96	0.00	0.01	0.00	0.02	4.19^b^	4.12	4.42^b^	4.45	17.69^a^^b^	63.62^a^^b^	14.79^a^^b^	49.31^a^^b^
120	0.00	0.00	0.00	0.00	4.31	3.89	4.48	4.33	19.28^a^^b^	73.06^a^^b^	16.83^a^^b^	59.91^a^^b^

Values represent averages from 5 experiments initiated on different cultures and on different days. N_2_O amounts were normalized to OD of each culture at each time point. Statistically significant differences between treatments were determined by Student's t-test at p < 0.05 and are designated as follows:

a *Significant difference between 0.3 and 1 mM NaNO_2_ treatment groups of wildtype (WT) or norZ_ch_ mutant cultures of S. wittichii RW1 at each time point*.

b *Significant difference between WT and norZ_ch_ mutant S. wittichii RW1 cultures incubated with 0.3 mM NaNO_2_ or 1 mM NaNO_2_*.

We next tested whether lower oxygen had an effect on the rates of NO^−^_2_ or O_2_ consumption or N_2_O production in non-mutated *S. wittichii* RW1. To address this question, *S. wittichii* RW1 cultures were inoculated with 0, 0.3, or 1 mM NaNO_2_ at either atmospheric or reduced (ca. 3%) O_2_ levels. Cultures initiated at atmospheric O_2_ and 1 mM NaNO_2_ consumed O_2_ and NO^−^_2_ significantly faster than cultures initiated at reduced O_2_ and 1 mM NaNO_2_, yet the rate of N_2_O production was 2–3 times faster for cultures initiated at reduced relative to atmospheric O_2_ levels (Table 4). The N_2_O-N measured in the gas headspace of the cultures was orders of magnitude lower than the amount of NO^−^_2_ consumed per unit biomass (i.e., nmol N_2_O produced from μmol NO^−^_2_ consumed). Even though N_2_O is highly soluble, the vast difference between NO^−^_2_ consumption and N_2_O production implies conversion of NO^−^_2_ into a product other than N_2_O; however, NO^−^_3_ was undetectable and NH^+^_4_ levels did not vary in any culture at any time point (data not shown).

**Table 4:** **Maximum rates of nitrite and oxygen consumption and nitrous oxide production by stationary phase *S. wittichii* RW1 wildtype cultures grown at atmospheric (ca. 22%) or reduced (ca. 3%) O_2_ headspace**.

**NaNO_2_ (mM) added to growth medium**	**Rate of O_2_ consumption (% headspaceOD^−1^ h^−1^)**		**Rate of NO^−^_2_ consumption (μmolOD^−1^ h^−1^)**		**Rate of N_2_O production (nmolOD^−1^ h^−1^)**	
	**Atmos. O_2_**	**Red. O_2_**	**Atmos. O_2_**	**Red. O_2_**	**Atmos. O_2_**	**Red. O_2_**
0	0.03	0.02	0	0	0	0
0.3	0.03	0.02	0.9	0.9	5.2^*^	16.1^*^
1.0	0.04^*^	0.02^*^	2.2^*^	1.5^*^	13.9^*^	29.8^*^

Values represent slopes of averaged measurements from 5 experiments initiated on different cultures and on different days (y-axis) over time (48–72 h; x-axis). The “

* *” indicates a significant difference between incubations initiated at atmospheric or reduced (3%) O_2_ headspace as determined by Student's t-test (p < 0.05). Calculated rates of were normalized to OD units of the cultures due to the difference in maximum biomass between cultures (Table 2)*.

### Expression levels of putative aerobic denitrification genes

**Figure 2:**
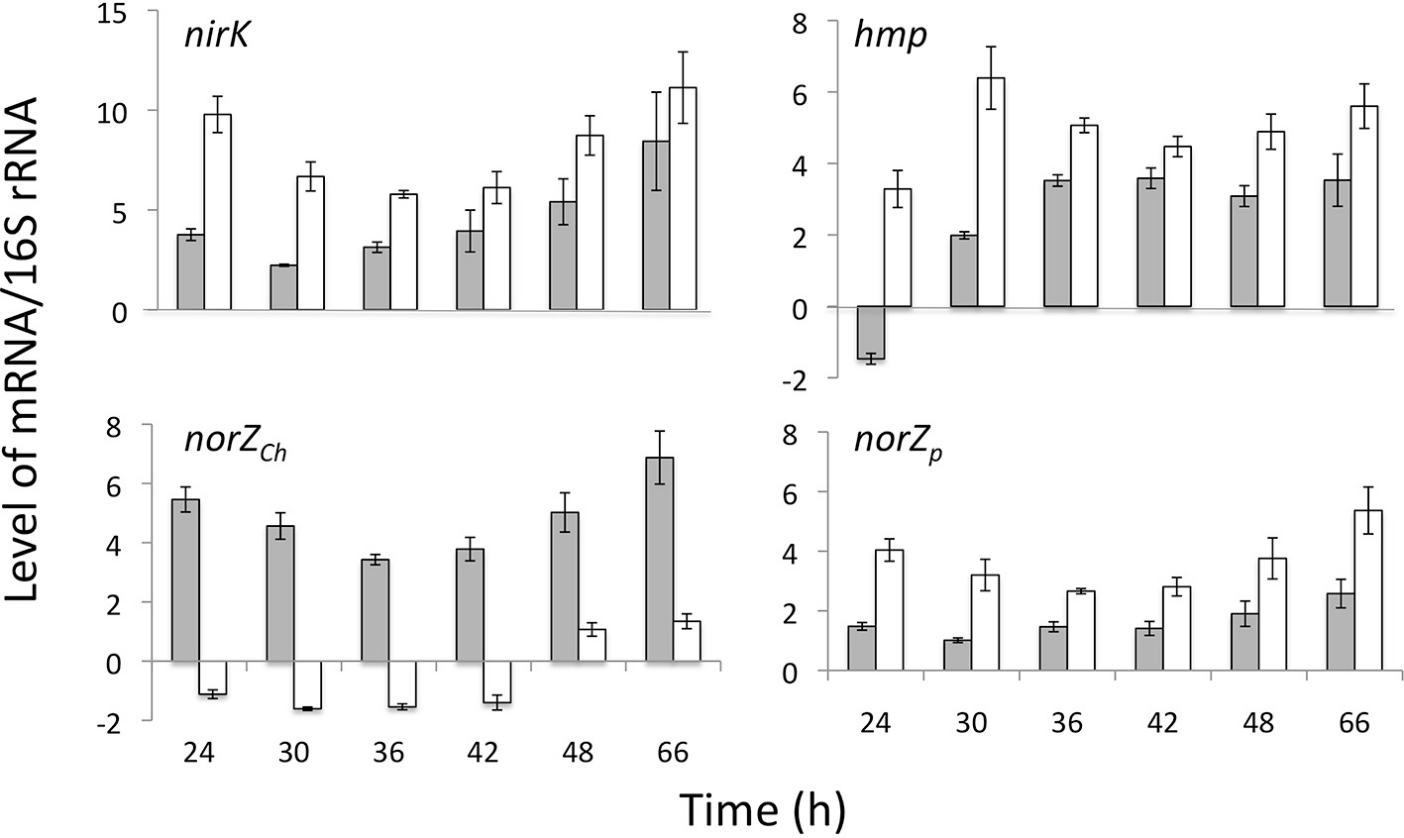
**Levels of mRNA of select genes in *S. wittichii* RW1 WT (gray bars) and *norZ*_ch_ mutant (white bars) in batch cultures over time**. Error bars represent standard error for 5 replicate experiments performed with separate cultures on different days.

Levels of specific mRNAs encoding *nirK*, *hmp*_p_, *norZ*_ch_, and *norZ*_p_, as normalized to levels of 16S rRNA, were compared between WT and *norZ*_ch_ mutant *S. wittichii* RW1 from mid-log and into stationary phase (24–66 h). This period of time covers the interval over which consumption of NO^−^_2_ and production of N_2_O is measurably active. Expression of *norZ*_p_ substituted for *norZ*_ch_ in the *norZ*_ch_ mutant strain and the levels of respective *norZ* transcript remained relatively high in both cell lines over time (Figure 2). Levels of *nirK* and *hmp*_p_ transcript were significantly higher in the *norZ*_ch_ mutant than in the WT strain at nearly all time points. Whereas *nirK* and *hmp*_p_ transcript levels increased between 24 and 66 h in WT cells, both transcript levels remained relatively high in the *norZ*_ch_ mutant over the full time course (Figure 2).

Finally, the effect of NO^−^_2_ on transcript levels was examined in *norZ*_ch_ mutant and WT cultures, and for non-mutated cultures initiated under atmospheric and reduced O_2_ levels. Each hybridization signal was normalized to that for 16S rRNA, after which the ratio of hybridization intensity between NaNO_2_-treated and untreated sample was calculated for every culture and each transcript pool. There was no significant effect of NO^−^_2_ treatment on any transcript level for any culture until late log phase (i.e., 24 h for WT and *norZ*_ch_ mutant cultures and 20 h for WT cultures initiated at reduced O_2_). At least a two-fold increase between NaNO_2_-treated and untreated cells was considered a significant effect; thus, *nirK* and *hmp_p_* were positively responsive to NaNO_2_ in both WT and *norZ*_ch_ mutant cultures (Table 5). For both *norZ* genes, only transcription levels of *norZ*_p_ in the *norZ_ch_* mutant were responsive to 1 mM NaNO_2_ treatment.

**Table 5:** **Effect of nitrite and O_2_ on levels of specific transcripts of *S. wittichii* RW1 from mid-log and early stationary phase cultures**.

**mRNA**	**WT (atmospheric O_2_)**				**WT (reduced O_2_)**				***norZ_ch_* (atmospheric O_2_)**			
	**0.3 mM**		**1.0 mM**		**0.3 mM**		**1.0 mM**		**0.3 mM**		**1.0 mM**	
	**24 h**	**36 h**	**24 h**	**36 h**	**20 h**	**24 h**	**20 h**	**24 h**	**24 h**	**36 h**	**24 h**	**36 h**
*nirK*	1.68^a^	1.56^a^	**3.98^a^**	**2.17^a^^b^**	0.89^a^	0.96	**2.00^a^**	1.38	1.81^a^	**2.28^a^**	**4.53^a^**	1.56^a^^b^
*hmp*	1.23	**2.20**	1.24^b^	**2.52**	1.26^a^	1.47^a^	**3.89^a^**	**2.65^a^**	1.18^a^	**2.28**	**2.00^a^^b^**	**2.79**
*norZ_ch_*	1.37^b^	1.51^a^^b^	1.42^b^	1.00^a^^b^	0.80	0.93	1.15	1.14	*Not expressed in this strain*			
*norZ_p_*	0.88^b^	0.88^b^	0.89^b^	0.91^b^	1.09	1.31	1.63	1.69	1.39^a^^b^	1.70^b^	**2.21^a^^b^**	1.58^b^

Values represent the ratio of hybridization intensity between nitrite-treated and untreated cells, previously normalized to 16S rRNA levels, and averaged from 5 experiments initiated on separate cultures on separate days. Bold values indicate a two-fold or greater increase in transcript level between NaNO_2_-treated and untreated cells. Statistically significant differences between treatments were determined by Student's t-test at p < 0.05 and are designated as follows:

a *Significant difference between 0.3 and 1 mM NaNO_2_ treatment groups of wild-type (WT) or norZ_ch_ mutant cultures of S. wittichii RW1 at the same time point*.

b *Significant difference between WT and norZ_ch_ mutant cultures initiated at atmospheric O_2_, incubated with 0.3 or 1 mM NaNO_2_*.

## Discussion

### *Sphingomonas wittichii* RW1 denitrifies NO^−^_2_ to N_2_O

Rapid consumption of NO^−^_2_ by *S. wittichii* RW1 occurred only once the cells reached stationary phase (Table 3), suggesting that *S. wittichii* RW1 performs this process for detoxification or maintenance metabolism rather than for generating proton motive force for cellular growth. During growth under reduced O_2_, an increased rate of NO^−^_2_ conversion to N_2_O (Table 4) relative to cultures initiated at atmospheric O_2_ implies that O_2_ limitation must be reached for denitrifying activity to commence as would be commonly expected (Zumft, 1997). It is interesting that a faster rate of NO^−^_2_ consumption occurred for cultures initiated at atmospheric than at reduced O_2_in the presence of 1 mM NaNO_2_ as this implies an additional process from denitrification for NO^−^_2_ loss. Although a substantial quantity of NO^−^_2_ consumed by *S. wittichii* RW1 was converted to N_2_O, there was a considerable pool of transformed NO^−^_2_ that could not be accounted for in NH^+^_4_ or NO^−^_3_ pools. There is no homolog for nitrous oxide reductase (*nosZ*) in the genome sequence of *S. wittichii* RW1; hence, denitrification to N_2_ is unlikely. Sphingomonads are also not known to produce N-storage polymers, but *S. wittichii* RW1 does encode an assimilatory nitrite reductase (*nirBD*; Swit_1707-8). Thus, the fate of the remaining NO^−^_2_-N remains unknown.

### Genes for nitrogen oxide transformations are expressed in *S. wittichii* RW1, and the *norz* genes are isofunctional

Levels of *nirK* and *hmp*_p_ and either *norZ*_ch_ (WT) or *norZ*_p_ (*norZ*_ch_ mutant) transcripts remained relatively high through stationary phase of *S. wittichii* RW1 (Figure 2), supporting the stationary phase onset of denitrifying activity (Table 3). The absence of *norZ_ch_* expression in *S. wittichii* RW1 had the effects of increasing the exponential growth rate and preventing slowed growth upon exposure to NO^−^_2_ (Figure 1 and Table 1). This phenotype may be in part due to increased expression of genes for handling nitrosative stress, that is *nirK*, *hmp*_p_, and *norZ*_p_, in the *norZ*_ch_ mutant compared to the WT (Figure 2). The increase in transcript pools corresponded to a decrease in the amount of NO^−^_2_ converted to N_2_O (Table 3), further suggesting that the *norZ*_ch_ mutant cells were not as susceptible to nitrosative stress as the WT. While other unexamined genetic factors were likely at play in mediating these phenotypes of the *norZ*_ch_mutant, the present data clearly show that the loss of *norZ*_ch_ expression was compensated for by expression of *norZ*_p_; hence, the *norZ* genes of *S. wittichii* RW1 are isofunctional. As with the WT cells, expression of both *nirK* and *hmp*_p_ genes were positively affected by exposure to NO^−^_2_ in the *norZ*_ch_ mutant (Table 5). This increased expression was potentially a function of *nirK* and *hmp*_p_ genes being regulated by NsrR (Swit_1789) and NnrR (Swit_5204) NO responsive regulators, respectively. In addition, the increased level of *norZ*_p_ transcript in the *norZ*_ch_ mutant upon exposure to 1 mM NaNO_2_ (Table 5), suggests a conditional co-regulation of *hmp-orf1-orf2-norZ* genes when *norZ*_p_ expression is required.

## Conclusions

Results from this study confirm the ability of *S. wittichii* RW1 to reduce NO^−^_2_ to N_2_O and also to transform excess NO^−^_2_ via another mechanism. This metabolic capability may be restricted to the *Spingomonas wittichii* species of the sphingomonads based on the limited co-occurrence of *nirK* and *norZ* genes in their genomes. This denitrification module was likely acquired by *S. wittichii* strains by lateral gene transfer as a function of ecological niche and need for N-oxide detoxification. As meta-'omic studies often rely on correlating functional genes to 16S rRNA phylotypes, this study sheds light on the complication of relatively rare LGT events that can confer biogeochemically important functions to individual species of broadly distributed bacterial families.

### Conflict of interest statement

The authors declare that the research was conducted in the absence of any commercial or financial relationships that could be construed as a potential conflict of interest.
